# What Is Sleep?

**Published:** 1920-10-02

**Authors:** 


### THE PUBLIC WELL-BEING.

### What is Sleep?

### ITS RELATION TO FATIGUE.

An old but interesting question was dealt with very recently by R. G. Gordon in a paper on "The Nature of Insomnia in the Psychoneuroses," contributed to the *Journal of Neurology*. What is insomnia? What is sleep? When we know all about sleep we shall know something which may bring good health and inexpressible peace to hundreds of thousands of suffering human beings. In dealing with insomnia it is essential, as the author of the paper points out, to get a clear idea as to what constitutes normal sleep; otherwise bad advice, based on ill-conceived ideas, may be given to the patient, with the result that his last state may be worse than his first.

"For example, it is common to hear a neurotic told to tire himself out and he will get sleep. Apart from the fact that, in spite of extreme fatigue, the patient often does not sleep, the person who gives this advice has not considered whether the sleep which is the result of fatigue is the same as natural sleep, or whether it is of the same benefit to the patient.

"Much has been written on the subject of sleep, but no very satisfactory definition has yet been arrived at. It would seem to be the result of a withdrawal of vital energy from the higher levels of consciousness, so that they no longer express themselves as conscious processes. The deeper the sleep, the more extensively is the content of what goes to make up the personality thrown out of action, so that in the deepest sleep only the vital reflexes persist, and of these we are seldom actively conscious. In lighter sleep the intellectual, rational, and controlling levels of consciousness are in abeyance, but the innate impulses and the constellations of ideas repressed into the lower levels of consciousness find expression in the form of dreams.

"How this withdrawal of energy takes place we do not know. Certain theories have been advanced, such as the theory of the retraction of the dendrites, but this sort of thing is necessarily purely speculative, and therefore without much value. We can only accept the fact that there is such a withdrawal of energy from consciousness, and study the conditions under which it is brought about. It would seem permissible to recognise three distinct sets of conditions: (1) Sleep induced under the influence of drugs; (2) sleep induced by fatigue; (3) natural sleep."

So far as the second form of sleep is concerned, many people would say that we sleep when we are tired and because we are tired, and while this is undoubtedly true it is not the only reason or even the usual one; we must remember that fatigue may be either physical or mental. Pure physical fatigue, the result of excessive bodily exercise, undoubtedly induces sleep. Personal experience of bodily fatigue during the war has proved that to most of us. Sleep under such circumstances was not only possible but imperative, even under the most disturbing conditions. Sleep, then, follows physical fatigue, and it also follows mental fatigue. In considering mental work we have to reckon with its ideational and emotional sides. In practice these cannot be divorced, for existence of completely feelingless thought has yet to be proved, and emotions cannot exist without ideas to which they attach themselves, although it is not necessary for these ideas to be conscious. The more highly toned with emotion a set of ideas may be, the more fatigue will be induced by its presence in consciousness. It is certainly more tiring to attend to a highly dramatic play or listen to emotional music than to add up columns of figures; and, as has been said, if too much work of this description is performed sleep will result; but this type of sleep would appear to be due also to a poisoning of the central nervous system, not by exogenous poisons, but by the endogenous products of fatigue which result from the metabolism of the cells of the nervous and muscular systems.

When we come to deal with natural sleep we must recognise that this is not the result of fatigue, but a protection against fatigue; that is, that it is not the result of harmful influences, but a biological protective reaction essential to the well-being of the individual. Boris Sidis, in his "Experimental Study of Sleep," shows clearly how sleep saves the organism from fatigue, and Bruce has amplified his conclusions that natural sleep is induced by monotony. The latter has pointed out that the more varied the mental equipment an individual possesses —that is, the greater the intellectual capacity which he enjoys—the less sleep he takes and the less he needs. The converse of this is quite obvious, for both young children and mental deficients, whose intellectual outlook is narrow and consequently monotonous, require and take a large amount of sleep. The same seeking after monotony is obvious in the steps which the ordinary person takes when he goes to bed. First he takes care to avoid physical distractions which might afflict him through his common sensations. This he achieves by lying in a comfortable bed, obtaining an equable temperature by adequate covering, and a pure atmosphere by adequate ventilation. Next he avoids extraneous mental distractions which might afflict him through his special senses, more especially of sight and hearing, by excluding light and sound. Finally, he completes the monotonous restriction of his consciousness by not paying attention to any intrinsic thoughts which enter his mind.

There is good reason to consider attention as the conative aspect of the libido or general vital energy, and therefore this deflection of attention from the stream of consciousness corresponds to the conception of sleep, as a withdrawal of libido from the higher levels of consciousness.

